# Subacromial bursa augmentation in arthroscopic rotator cuff repair: Clinical and doppler ultrasound assessment—A preliminary study

**DOI:** 10.1002/jeo2.70714

**Published:** 2026-05-12

**Authors:** Florian Freislederer, Mara Dimitriu, David Endell, Laurent Audigé, Daniela Brune, Marco Etter, Markus Scheibel

**Affiliations:** ^1^ Department of Shoulder and Elbow Surgery Schulthess Klinik Zürich Switzerland; ^2^ Department of Research and Development Schulthess Klinik Zürich Switzerland; ^3^ Department of Clinical Research University Hospital Basel and University of Basel, Surgical Outcome Research Center Basel Switzerland; ^4^ Department of Rheumatology Schulthess Klinik Zürich Switzerland; ^5^ Charité‐Universitaetsmedizin Berlin, CMSC Center for Musculoskeletal Surgery Berlin Germany

**Keywords:** bursa augmentation, rotator cuff repair, tendon healing, tendon thickness, ultrasound Doppler

## Abstract

**Purpose:**

To determine whether augmentation with the subacromial bursa influences postoperative tendon healing, particularly regarding morphology and vascularity, after arthroscopic rotator cuff repair.

**Methods:**

In this prospective comparative study (2019–2023), patients with crescent‐shaped posterosuperior rotator cuff tears (SSP ± ISP, Lafosse ≤ 1), tendon retraction Patte ≤ 2, and fatty infiltration Goutallier ≤ 2 underwent double‐row arthroscopic repair with (Bursa Group) or without (Control Group) bursa augmentation. Postoperative vascularity and tendon integrity were assessed via Doppler ultrasound at 3, 6, 12 and 24 weeks. Secondary outcomes included pain and functional scores.

**Results:**

Thirty patients (mean age 60) were analysed: 20 in the Bursa Group and 10 in the Control Group. The Bursa Group showed an initial increase in bursal vascularity at 3 weeks, which declined similarly in both groups thereafter. At 6 weeks, neovascularity at the tendon insertion showed an increase in the Bursa Group, while it significantly decreased in controls (*p* = 0.032). Pain scores were lower in the Bursa Group, with statistically significant but small differences at 6 weeks at rest (mean VAS 1 ± 1 vs. 3 ± 2, *p* = 0.013) and at 3 months during activity (mean VAS 3 ± 2 vs. 5 ± 2, *p* = 0.009), while no significant differences were observed at other time points. Tendon thickness was better preserved in Group B at 3 (*p* = 0.029) and 6 months (*p* = 0.007). Functional outcomes were comparable after 6 months. Three retears occurred in Group B, while no retears were observed in Group C.

**Conclusion:**

Clinical outcomes were similar between bursa‐augmented rotator cuff repair and the control group. While bursa augmentation did not enhance neovascularization compared to controls, it was associated with better tendon thickness preservation and reduced pain, although these differences were marginal and limited to selected early follow‐up time points.

**Level of Evidence:**

Level III, evidence study.

## Graphical abstract

Bursa augmentation in arthroscopic rotator cuff repair preserved tendon thickness and reduced early postoperative pain without increasing neovascularization.

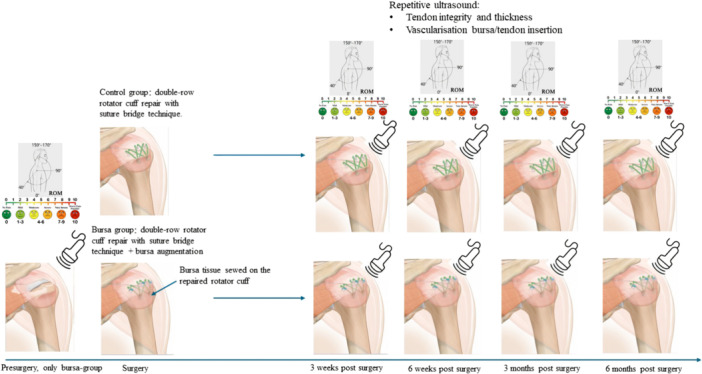

**Abbreviations**
ARCR: arthroscopic rotator cuff repair
ASA: American Society of Anesthesiologists (Score)
BMI: body mass index
C‐shaped: crescent‐shaped
Group B: bursa augmentation group
Group C: control group
IL‐1: interleukin 1
IL‐6: interleukin 6
ISP: infraspinatus
L‐PRF: leucocyte‐ and platelet‐rich fibrin
MCID: minimal clinically important difference
MMP‐1: matrix metalloproteinase 1
MMP‐9: matrix metalloproteinase 9
MSC: mesenchymal stem cells
NRS: numeric rating scale
PDS: polydioxanone sutures
PRP: platelet‐rich plasma
ROI: region of interest
ROM: range of motion
SD: standard deviation
SSC: subscapularis
SSP: supraspinatus
TNF‐alpha: tumour necrosis factor alpha
VEGF: vascular endothelial growth factor

## INTRODUCTION

Rotator cuff tears are among the most common shoulder injuries, with a prevalence of up to 30% in the general population [31]. Despite advances in surgical techniques, retear rates remain high—approximately 14% overall and up to 40% in large or massive tears [10, 27, 32]. Since mechanical improvements in suture configuration have not significantly reduced these rates [3], biological strategies to enhance tendon healing have become a major research focus.

Several biological augmentation approaches have been explored, including growth factors, platelet‐rich plasma, and acellular dermal allografts. While some of these methods have shown potential benefits, their clinical results remain inconsistent, and issues such as cost and immune response limit their routine application [18, 25, 26, 29].

The subacromial bursa, traditionally regarded as a source of pain and inflammation [2, 36, 37] has recently gained attention as a biologically active tissue that may support tendon repair. It provides rich vascularisation and contains mesenchymal stem cells capable of differentiating into tenogenic and angiogenic lineages [5, 30, 34]. These regenerative properties suggest that preserving or augmenting the rotator cuff repair with bursal tissue could enhance revascularization and tendon healing [1, 8, 20].

The objective of this study was to determine whether augmentation with subacromial bursa tissue influences postoperative tendon morphology and vascularity after arthroscopic rotator cuff repair. We hypothesised that the augmented bursal tissue would adhere to the repaired tendon and enhance peritendinous vascularisation, thereby supporting tendon healing.

## METHODS

### Study population

This study was a prospective, quasi‐experimental, non‐randomised comparative single‐center study with consecutive enrolment of eligible patients between October 2019 and August 2023. The surgical procedures were performed by five shoulder specialised surgeons with extensive experience in arthroscopic rotator cuff repair. Patients with Crescent‐shaped (C‐shaped) posterosuperior rotator cuff tear treated by arthroscopic double row rotator cuff repair using the suture bridge technique were enroled and included if intraoperative observations confirmed that performing additional bursa augmentation intervention could be performed. We included patients with C‐shaped tears of the supraspinatus (SSP) and infraspinatus (ISP) tendons. If a subscapularis (SSC) tendon tear was present, it had to be classified as Lafosse ≤ 1 [15], requiring only debridement. Exclusion criteria were patients with a tendon retraction ≥ 2 according to Patte [24], fatty infiltration of the supraspinatus tendon ≥ 2 according to Goutallier [11], subscapularis tear Lafosse > 2, the presence of systematic rheumatologic‐inflammatory disease and/or diabetes mellitus, injection history (subacromial cortisone infiltration ≤ 6 months) and smoker.

Eligible patients were recruited consecutively, with the first enroled 20 patients receiving and additional bursa augmentation (Group B) and the following 10 patients not receiving that intervention for the control group (Group C).

Patients in whom bursal augmentation was not technically feasible were excluded after intraoperative evaluation, as the quality of the bursal tissue (e.g., fragile) was considered a critical prerequisite for the intervention. Therefore, these cases were not included in the control group to avoid confounding by poor tissue quality.

This study was performed according to the ethical standards of the Declaration of Helsinki and all included patients provided written consent. The study was approved by the Ethics Committee of the Canton Zürich. (approval no. 2019‐01073; date of approval: 30 July 2019).

### Ultrasound and functional assessments

Preoperative ultrasound was performed in the Bursa Group only as a baseline assessment of the tendon and bursal tissue intended for augmentation; the Control Group did not undergo preoperative ultrasound because the primary study focus was postoperative vascularity and structural evolution over time. All patients underwent ultrasound examination after 3 and 6 weeks and 3 and 6 months. Surgical data and clinical data at baseline and after 6 months were retrieved from the local rotator cuff registry [7].

The examinations were performed by four sonographers with extensive experience in musculoskeletal ultrasound, particularly in postoperative evaluation of rotator cuff repair. All sonographers are certified in musculoskeletal ultrasound and followed the same standardised scanning protocol to ensure consistency across examinations (Supporting Information: Additional File 1). Each examiner performs approximately 900 shoulder ultrasound examinations per year; however, interobserver reliability testing was not performed. All examinations were performed with a Philips Epiq machine with a multifrequency linear transducer (7–15 MHz) in B mode. Sonographers were not formally blinded to group allocation because surgical history was accessible in the clinical record; however, they were not explicitly informed about whether bursa augmentation had been performed before each examination. For the ultrasound examination, the patient was in a sitting, upright position in a chair with the arm hanging next to the body. Because of postoperative limitations of range of motion, the rotation and extension of the arm was adjusted to give the best possible view on the rotator cuff reconstruction and bursa‐augmentation site while guaranteeing best possible patient comfort. For evaluation of tendon and bursa‐augmentation integrity, longitudinal and transversal planes were examined and dynamic manoeuvres performed. The tendon‐thickness was measured at the osteo‐articular edge. Tissue beginning directly above the bone/cartilage (lower edge) up to the bursa augmentation, fluid or muscle tissue was counted as tendon, knowing, that these measurements included the capsule‐ligamentous complex as well as the tendon itself. For the grading of vascularisation, the same position was used, but the whole length and width of the reconstructed tendon was assessed and the stress on the tendon/tissue was reduced to a minimum (e.g. reduction of arm retroversion), since extensive stretching of the tendon might lead to vessel compression. Pulse repetition frequency was set to 0.5 kHz and the Doppler frequency to 2/3 of B‐mode. Gain was adjusted to get minimal/no noise (e.g. at cortical boarders) but maximal sensitivity. For grading we used the modified Öhberg score [22, 35]. It has not been specifically validated for postoperative rotator cuff evaluation, but tendon tissues, the peritendinous region, vascularisation, and bursal structures can be similarly visualised and assessed using ultrasound. The modified Öhberg score is a five‐level scale used to assess neovascularization in different tendinopathies. A score of 0 means no Doppler signal, 1+ indicates 1–2 new vessels in the Kager's fat pad, 2+ represents 1–2 vessels inside the tendon, 3+ corresponds to 3–4 intratendinous vessels, and 4+ denotes a dense network with more than 5 intratendinous vessels.

Patient‐experienced pain at rest and during activities using a numeric rating scale (NRS, 0 = no pain to 10 = worst pain) were also documented during the ultrasound examination.

Functional outcomes documented in the local rotator cuff registry included active range of motion (ROM) for anterior forward flexion in the scapular plane, abduction, and external rotation at 0° abduction and shoulder strength in 90° abduction. Internal rotation was documented by Apley's test categories. Postoperative complications were documented throughout the follow‐up period up to 6 months.

### Surgical procedures

**Figure 1:**
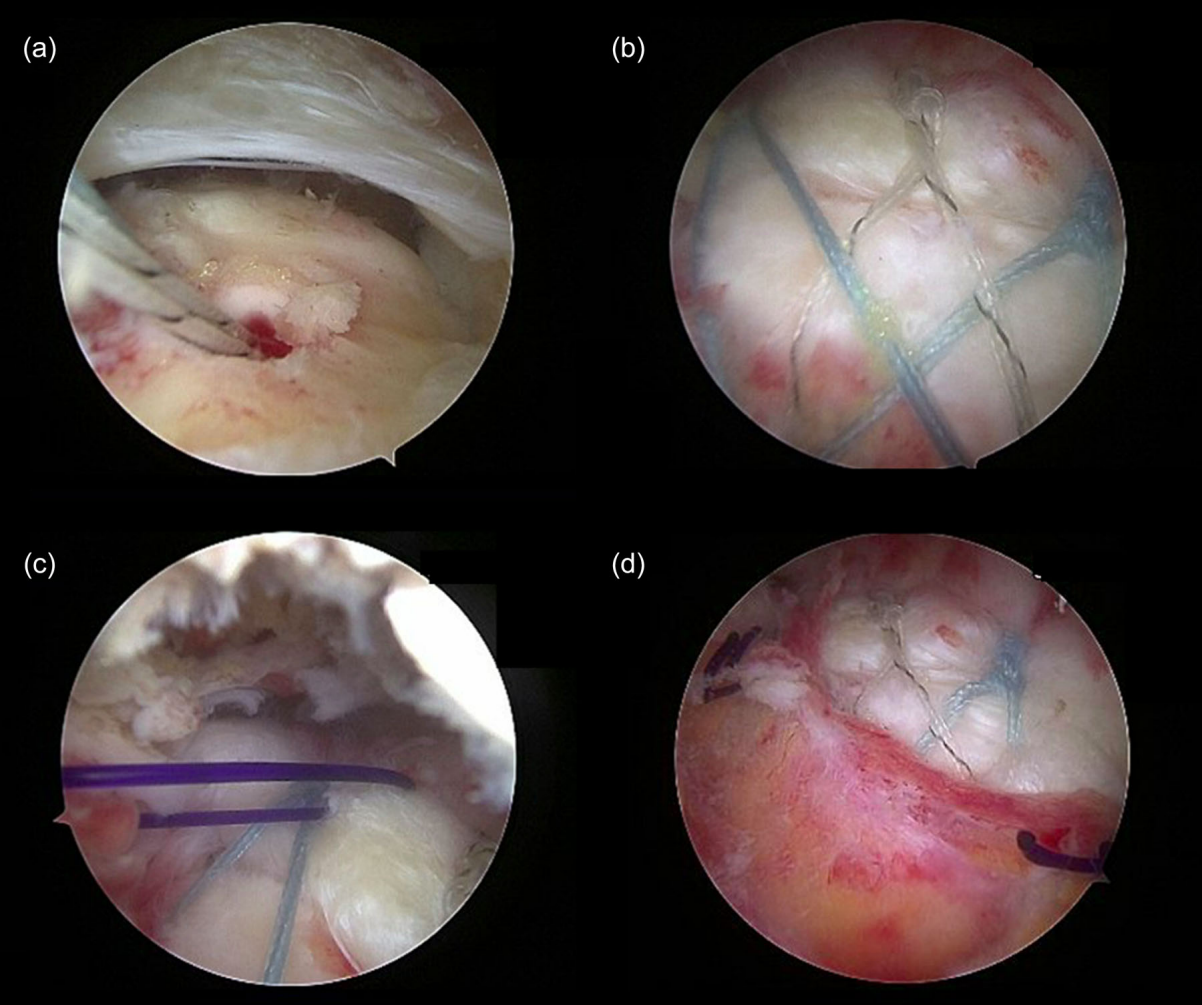
Arthroscopic views from the lateral portal of the subacromial space showing the bursa augmentation technique (a–d). (a): The rotator cuff tear (supraspinatus tendon lesion) is shown in the upper half of the image, and a suture anchor has been placed in the humeral footprint (centrally on the image). (b): The image demonstrates the repaired rotator cuff. (c): The subacromial bursa, visible from approximately the 8‐ to 10‐o'clock position, is looped with a polydioxanone (PDS) suture, mobilised, and drawn over the reconstructed rotator cuff (4‐ to 6‐o'clock position). (d): The subacromial bursa, now covering the reconstructed rotator cuff, is shown; at approximately the 4‐o'clock and 10‐o'clock position, the PDS suture used to secure the bursa is visible.

In this study, the surgical technique for the double‐row rotator cuff repair using the suture bridge technique with bursa augmentation was performed as extensively described in a previous publication [8]. In both groups, a double‐row rotator cuff repair was performed using the suture bridge technique. In Group B a bursa augmentation was additionally performed according to a previously published method [8]. After completing the rotator cuff reconstruction, the parietal layer of the subacromial bursa is carefully mobilised from the lateral subdeltoid region. This well‐vascularises and inflammation‐free bursal tissue is then sutured side‐to‐side onto the tendon using a suture‐lasso device and polydioxanone (PDS) sutures (Figure 1). Several sutures are placed posteriorly aiming to cover the largest possible portion of the tendon footprint (in this case the middle and lateral parts) with the bursal layer. Finally, an arthroscopic acromioplasty is performed to remove soft tissue from the acromion and increase the subacromial space.

### Postoperative rehabilitation

A standardised postoperative rehabilitation protocol was followed by all patients. The operated shoulder was immobilised in a sling in internal rotation for 6 weeks and only passive mobilisation was allowed during this period. After 6 weeks patients started to actively mobilise the shoulder and 3 months after surgery strength exercises were added to the protocol.

### Data management and statistical analysis

Study data and registry data were managed using the REDCap Electronic Data Capture system [14] and exported for statistical analysis using Stata version 17 (Stata Corp LP, College Station, TX). Baseline patient demographic, ultrasound and functional parameters were tabulated separately per group using standard descriptive statistics. The outcome parameters were also tabulated per group at baseline as well as at each one of the follow‐ups timepoints.

Non‐parametric tests were performed for within‐group differences (Wilcoxon‐signed rank test) and between‐group differences (Mann–Whitney *U* test) with a significance level set at 0.05.

## RESULTS

**Figure 2:**
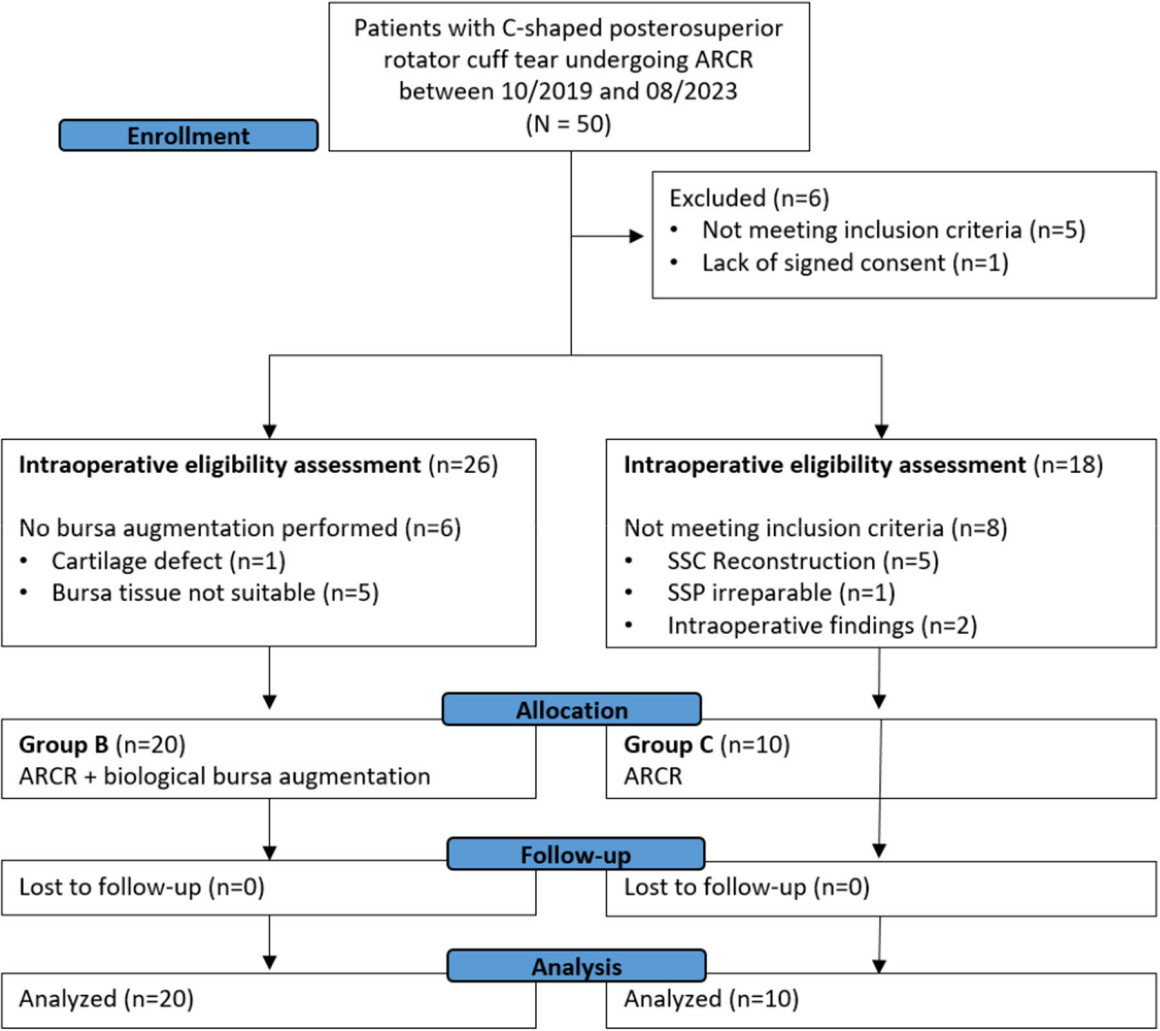
Flowchart of patient inclusion. ARCR, arthroscopic rotator cuff repair; SSC, subscapularis; SSP, supraspinatus.

A total of 50 patients were enroled during the study period. Of these, six were excluded prior to surgery because they did not meet inclusion criteria (*n* = 5) or due to lack of signed informed consent (*n* = 1). The remaining patients underwent intraoperative eligibility assessment and were allocated consecutively according to the surgical strategy. The first consecutive cohort was assigned to ARCR with bursa augmentation (Group B). In this cohort, six patients were excluded intraoperatively because bursa augmentation could not be performed because the texture of the bursa was not suitable for the intervention (e.g., too thin or fragile) or because of intraoperatively detected exclusion criteria. Subsequently, a second consecutively cohort underwent ARCR without bursa augmentation (Group C). In this cohort, 8 patients were excluded intraoperatively due to repair of subscapularis tendon (*n* = 5), irreparable supraspinatus tendon (*n* = 1), or intraoperative cartilage lesion > Grade 2 (*n* = 2) [23]. Thus, 30 patients were included for final data analysis with 20 patients in Group B and 10 patients in Group C (Figure 2).

### Patient demographics

Baseline demographic and clinical characteristics are presented in Table 1. The two groups were comparable in terms of sex distribution, age at surgery, dominant arm involvement, and body mass index, with no relevant between‐group differences.

**Table 1:** Baseline patient characteristics.

	Total *N* = 30	Group B *N* = 20	Group C *N* = 10
Age, M (SD)	60 (10)	60 (11)	61 (8)
Male sex, *N* (%)	16 (53)	12 (60)	4 (40)
Dominant side operated, *N* (%)	19 (63)	12 (60)	7 (70)
ASA‐classification, *N* (%)			
ASA I	5 (17)	3 (15)	2 (20)
ASA II	22 (73)	15 (75)	7 (70)
ASA III	3 (10)	2 (10)	1 (10)
BMI, M (SD)	28 (5)	29 (5)	27 (5)
Tear pattern, *N* (%)			
SSP	20 (67)	14 (70)	6 (60)
SSP & ISP	5 (17)	2 (10)	3 (30)
SSP & ISP & SSC	1 (3)	NA	1 (10)
SSP & SSC	4 (13)	4 (20)	0 (0)
Retraction of SSP tendon according to Patte, *N* (%)			
No retraction	6 (20)	5 (25)	1 (10)
Patte I	18 (60)	11 (55)	7 (70)
Patte II	6 (20)	4 (20)	2 (20)
Fatty infiltration of SSP tendon according to Goutallier, *N* (%)			
Level 0	14 (47)	9 (45)	5 (50)
Level I	12 (40)	9 (45)	3 (30)
Level II	4 (13)	2 (10)	2 (20)

Abbreviations: ASA, American Society of Anesthesiologists; BMI, body mass index; ISP, infraspinatus; SD, standard deviation; SSC, subscapularis; SSP, supraspinatus.

### Ultrasonography and functional outcomes

**Figure 3:**
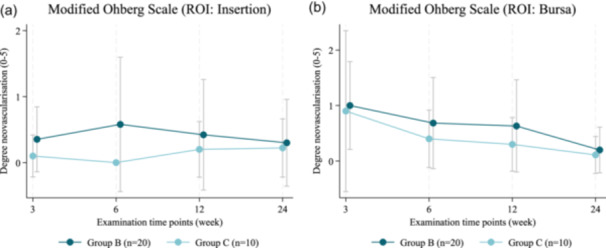
Modified Öhberg Scale. (a) Region of interest (ROI): reinserted tendon (supraspinatus). (b) ROI: Bursa. Group B: ARCR and bursa augmentation, Group C: ARCR without bursa augmentation. ARCR, arthroscopic rotator cuff repair.

Neovascularization scores in the bursa region did not differ significantly between Group B and Group C at any postoperative time point (Figure 3 and Supporting Information: Additional File 2). In the insertion region, median neovascularization scores were comparable between groups at 3 weeks, 3 months and 6 months. At 6 weeks, Group B demonstrated higher neovascularization scores than Group C (median 0 [range, 0–4] vs. 0 [range, 0–0]; *p* = 0.032).

**Figure 4:**
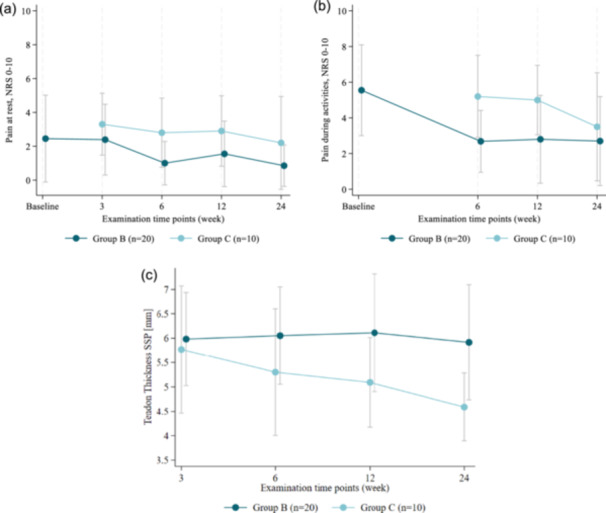
Ultrasonography outcomes. (a) Pain at rest (Numeric Rating Scale (NRS): 0–10). (b) Pain during activities (0–10). (c) Supraspinatus tendon thickness (mm). Group B: ARCR and bursa augmentation, Group C: ARCR without bursa augmentation. ARCR, arthroscopic rotator cuff repair.

Mean pain outcomes at rest and during activities are shown in Figure 4a,b. At 6 weeks, Group B reported lower pain scores than Group C both at rest (median 0 [range, 0–4] vs. 2 [range, 0–6]; *p* = 0.013) and during activities (median 1 [range, 0–6] vs. 6 [range, 2–8]; *p* = 0.006). At this time point, median pain scores in Group B were within ranges corresponding to patient acceptable symptomatic state (PASS ≤ 2) and substantial clinical benefit (SCB ≤ 1) as reported for 0–10 pain scales after arthroscopic rotator cuff repair, whereas median scores in Group C exceeded these thresholds. At 3 months, activity‐related pain remained lower in Group B (median 2 [range, 0–9] vs. 4 [range, 3–8]; *p* = 0.009), whereas pain at rest did not differ significantly between groups (*p* = 0.078).

Supraspinatus tendon thickness did not differ significantly between groups at 3 or 6 weeks. At 3 and 6 months, median tendon thickness was greater in Group B than in Group C (3 months: median 6 mm [range, 4–8] vs. 5 mm [range, 4–7], *p* = 0.029; 6 months: median 6 mm [range, 4–8] vs. 5 mm [range, 3–5], *p* = 0.007) (Figure 4c). These differences were sub‐millimetric and should be interpreted with caution given the expected variability of ultrasonographic measurements.

At the final follow‐up at 6 months, ultrasonography detected partial supraspinatus tendon ruptures in two patients in Group B, and one patient had both a partial supraspinatus and a complete infraspinatus rupture requiring surgical repair. There were no indications of tendon rupture among patients in Group C 6 months after their surgeries.

No significant postoperative functional improvement at 6 months compared to baseline was observed in the overall cohort (Table 2).

**Table 2:** Functional outcomes for both groups.

	Group B			Group C			
Parameters	*n*	Mean (SD)	Median (range)	*n*	Mean (SD)	Median (range)	*p*‐value
Active flexion (°)							
Baseline	20	153 (24)	160 (90–180)	10	158 (9)	160 (140–170)	0.891
6 Months	20	159 (13)	160 (110–170)	10	149 (17)	155 (120–170)	0.078
Active abduction (°)							
Baseline	20	137 (34)	145 (70–180)	10	156 (13)	160 (130–170)	0.244
6 Months	20	149 (21)	150 (90–170)	9	151 (15)	150 (130–170)	0.828
Active external rotation in 0° abduction (°)							
Baseline	19	60 (13)	60 (35–80)	10	62 (12)	65 (40–80)	0.776
6 Months	20	62 (14)	65 (30–80)	10	63 (13)	68 (40–80)	0.854
Strength in abduction (kg)							
Baseline	20	5.1 (2.6)	5.0 (0.0–10.0)	8	5.3 (1.8)	5.5 (2.9–7.5)	0.818
6 Months	20	6.5 (2.6)	6.5 (1.0–12.0)	7	5.7 (1.6)	5.0 (4.0–9.0)	0.161

*Note*: *p*‐value = Two‐sample Wilcoxon rank‐sum (Mann–Whitney) test for continuous variables.

Abbreviation: SD, standard deviation.

## DISCUSSION

The aim of this study was to investigate the effect of bursa augmentation in arthroscopic rotator cuff repair. Our main finding was that bursa augmentation led to marginally thicker tendons over a 6‐month follow‐up, whereas the control group experienced a decrease in tendon thickness. Clinically, pain at rest and during activities tended to be higher in the group without augmentation, but statistical significance was reached only at 6 weeks (pain at rest) and 3 months (pain during activities) (*p* = 0.009). We did not observe a relevant increase in intratendinous neovascularization with bursa augmentation. No cases of frozen shoulder were observed, and functional outcomes were similar between groups. Two partial retears occurred in the bursa group and one complete traumatic infraspinatus rupture required revision surgery in a patient that was treated with a supraspinatus repair + bursa augmentation, while no retears were detected in the control group at 6 months. Overall, these findings suggest that bursa augmentation does not have a deleterious effect on early clinical outcomes.

The results of this study support a potential beneficial role of bursa augmentation, which is further reflected in recent clinical studies. Gregory et al. reported that reimplanted bursal tissue did not negatively affect clinical outcomes, with retear rates comparable to those observed in the control group [12]. Similarly, Güler et al. found that combining bursa augmentation with acromioplasty in patients with partial‐thickness rotator cuff tears led to improved clinical outcomes and a significantly lower retear rate [13]. A more recent study demonstrated that even in patients with massive rotator cuff tears, bursa augmentation resulted in significantly better functional and pain outcomes, with no retears detected on ultrasound one year postoperatively in a cohort of 48 patients [6]. These findings challenge the traditional view of the subacromial bursa in rotator cuff disease as purely inflammatory tissue due to the presence of proinflammatory mediators [37], suggesting a potential regenerative role once structural pathologies are addressed. This aspect was further highlighted in a narrative review by Ma et al., which concluded that, despite its association with inflammatory pain in patients with rotator cuff disease, the subacromial bursa contains biologically active mediators and mesenchymal stem cells that may support tendon regeneration and improve outcomes [19]. Our hypothesis is that the inflammatory activity of the bursa may be context‐dependent and subsides once the tendon is repaired.

Regarding tendon thickness, previous ultrasound and MRI studies on conventional rotator cuff repair have reported either a decrease or no significant change in tendon thickness after repair, with limited correlation to clinical outcomes [16, 33, 39]. Our findings suggest that bursa augmentation may help preserve tendon structure, even increasing thickness although the clinical significance remains uncertain, especially given the small sample size and short follow‐up. An alternative interpretation of the observed increase in tendon thickness may be persistent postoperative inflammatory activity rather than enhanced tendon healing. However, no clinical signs of increased pain, stiffness, or functional impairment were observed at 6 months, arguing against a clinically relevant inflammatory response.

Sonographic evaluation of vascularity revealed only transient peribursal neovascularization at 3 weeks postoperatively in the augmented group, which subsequently decreased to levels comparable to the control group. These findings align with previous studies showing minimal intratendinous perfusion after rotator cuff repair indicating that bursa augmentation does not substantially modify vascularisation in the mid‐term [4, 9, 33, 39].

In our study, the follow‐up period was limited to 6 months, which may be considered short for assessing long‐term tendon healing. However, previous research indicates that most retears occur within this early postoperative phase. Miller et al. reported that, among 22 patients with large rotator cuff tears, seven of nine retears occurred within the first 3 months and two within 6 months, with no additional retears detected thereafter [21]. Similarly, a recent systematic review by Longo et al. demonstrated that more than half of all retears develop within the first few postoperative months [17]. In a large cohort study by Yamaura et al., including 638 surgically treated shoulders, 95.1% of the 44 retears occurred within the first postoperative year [38]. These findings suggest that a 6‐month follow‐up, as used in our study, captures the critical period for retear occurrence, although longer‐term assessments remain valuable for evaluating sustained tendon integrity and functional recovery. Nevertheless, more recent evidence suggests that the temporal pattern of retears may be more heterogeneous than previously assumed, with clinically relevant failures occurring both early and later during follow‐up [28].

Overall, bursa augmentation appears to be a safe procedure that may support tendon integrity and reduce early postoperative pain without increasing the risk of stiffness. However, the short 6‐month follow‐up limits conclusions about long‐term tendon healing and retear rates, highlighting the need for extended follow‐up studies with imaging‐based evaluation.

### Limitations

This study has several limitations. First, the quasi‐experimental, sequential, and non‐randomised allocation of patients—assigning the first 20 eligible patients to the bursa augmentation group and the subsequent 10 to the control group—introduces potential selection bias and limits the internal validity of the results. The relatively small sample size further reduces statistical power and generalisability. In addition, blinding was not feasible, and postoperative functional assessments were performed by the treating surgeons, which may have introduced observer bias.

Even if the sonographic assessments were conducted by four experienced examiners, no interobserver reliability testing was performed, which may have led to variability in ultrasound findings. Magnetic resonance imaging (MRI) according to the Sugaya classification—the current gold standard for postoperative assessment of tendon integrity—was not performed; however, vascularity can be better visualised using Doppler ultrasound, which provides dynamic information on blood flow not captured by MRI. On the other hand, in our clinical setting, routine MRI follow‐up was not deemed cost‐effective from a health‐economic standpoint, making ultrasound a practical and efficient alternative for postoperative evaluation.

A relevant number of patient questionnaires required for the calculation of composite outcome scores (SSV, CS and OSS) were missing, allowing only partial analysis of these parameters. In addition, patients in whom bursal augmentation was not technically feasible were excluded rather than reassigned to the control group to avoid confounding by poor tissue quality, which may nonetheless introduce selection bias. Nevertheless, the 6‐month follow‐up period was relatively short and may limit the assessment of long‐term tendon integrity and clinical outcomes.

## CONCLUSION

Clinical outcomes were similar between bursa‐augmented rotator cuff repair and the control group. While bursa augmentation did not enhance neovascularization compared to controls, it was associated with better tendon thickness preservation and reduced pain, although these differences were marginal and limited to selected early follow‐up time points.

## AUTHOR CONTRIBUTIONS

**Florian Freislederer**: Data curation; investigation; methodology; project administration; validation; visualisation; major contributor in writing the manuscript. **Mara Dimitriu**: Major contributor in writing the manuscript. **David Endell**: Data curation; methodology. **Laurent Audigé**: Investigation; methodology; project administration validation; visualisation. **Daniela Brune**: Investigation; methodology; validation; visualisation. **Marco Etter**: Data curation; validation; vizualization. **Markus Scheibel**: Data curation; investigation; methodology; project administration; resources; validation; supervision.

## CONFLICT OF INTEREST STATEMENT

The authors declare no conflict of interest.

## ETHICS STATEMENT

This study was performed according to the ethical standards of the Declaration of Helsinki and all included patients provided written informed consent. The study was approved by the Ethics Committee of the Canton of Zurich. Ethic approval no. 2019‐01073. TRIAL Registration: NCT03986749, 11.06.2019.

## Supporting information

AdditionalFile1.

AdditionalFile2.

## Data Availability

The data that support the findings of this study are available on request from the corresponding author. The data are not publicly available due to privacy or ethical restrictions.
