# Supplementary material for: Subacromial bursa augmentation in arthroscopic rotator cuff repair: Clinical and doppler ultrasound assessment—A preliminary study
Source: J Exp Orthop. 2026 May 12;13(2):e70714. doi: 10.1002/jeo2.70714 (PMC13162131; doi:10.1002/jeo2.70714)
Supplement: Supplementary file 1 — AdditionalFile1. [file JEO2-13-e70714-s002.docx]

**Standardised ultrasound protocol:** to ensure comparability we predefined a standardises ultrasound protocol using 3 steps. All sonographers were briefed and trained in that protocol, that also represents standard postoperative ultrasound examination at our centre, hence every sonographer already had a broad experience with this protocol.

1. Tendon integrity assessment: patient sitting in upright position in a chair with the arm hanging next to the body. Rotation and extension of the arm were adjusted to give the best possible view on the rotator cuff reconstruction and bursa-augmentation site.

Figure 1: Longitudinal view on the reconstructed supraspinatus tendon

Figure 2: Transversal view on the reconstructed supraspinatus tendon

1. Measurement of tendon thickness


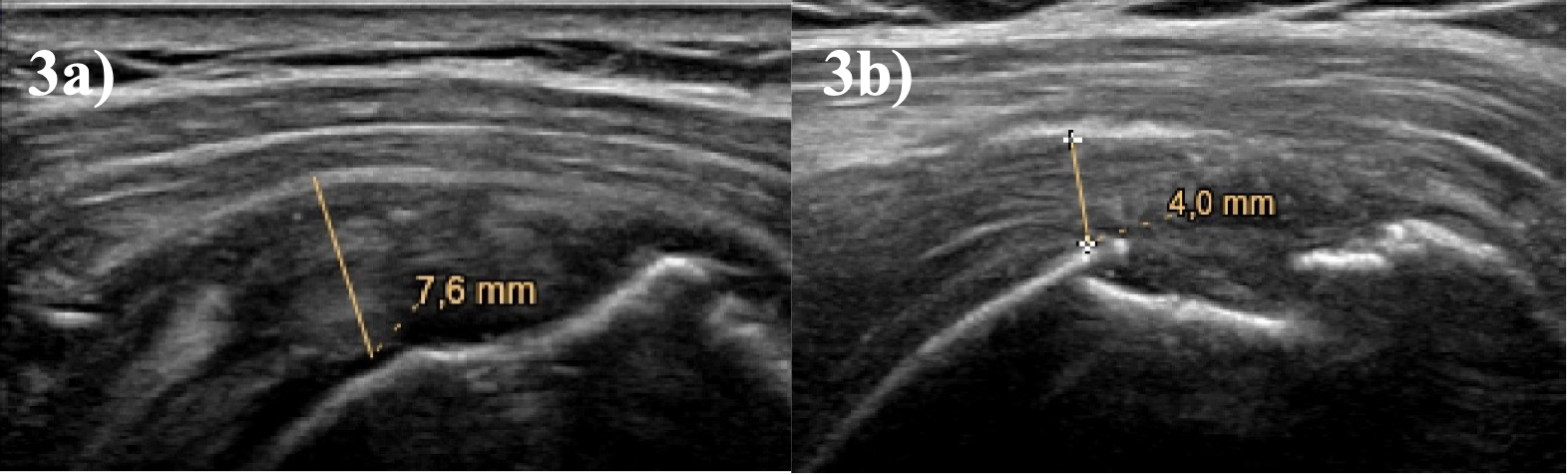


Figure 3 a: Measurement of tendon-thickness with bursa augmentation

Figure 3 b: Measurement of tendon-thickness without bursa augmentation

Assessment of hyperarization using Doppler sonography: for the grading of vascularization, the whole length and width of the reconstructed tendon was assessed and the stress on the tendon/tissue was reduced to a minimum (e.g. reduction of arm retroversion), since extensive stretching of the tendon might lead to vessel compression. Pulse repetition frequency was set to 0.5 kHz and the Doppler frequency to 2/3 of B-mode. Gain was adjusted to get minimal/no noise (e.g. at cortical boarders) but maximal sensitivity. For grading we used the modified Öhberg score.


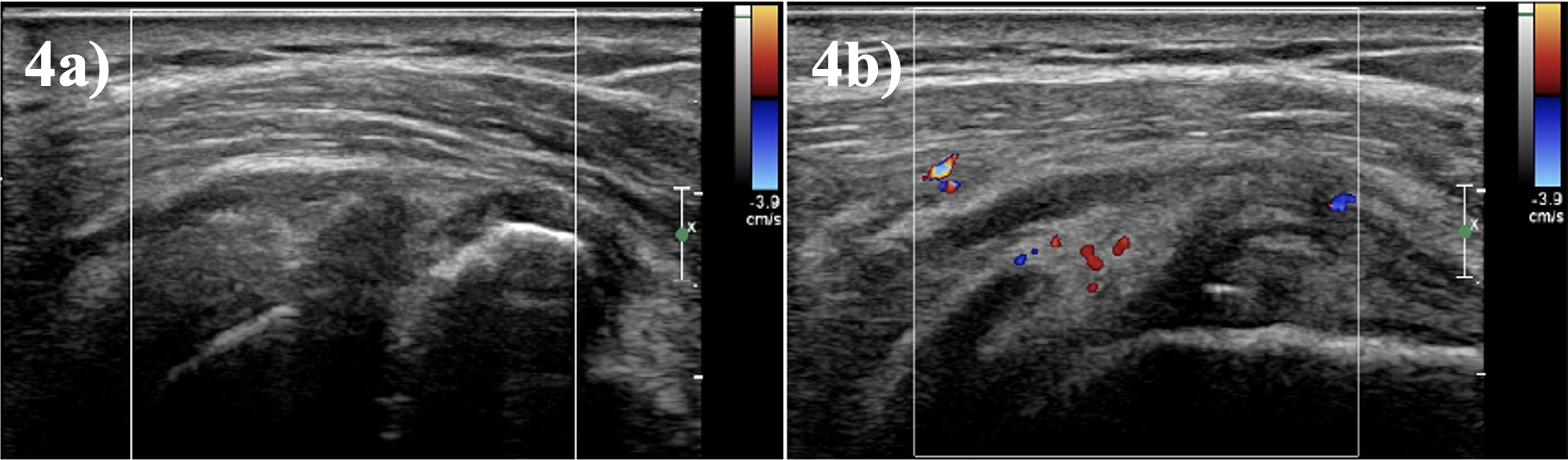


Figure 4 a: Vascularization grade 0 by modified Öhberg score

Figure 4 b: Vascularization grade 2 by modified Öhberg score (2 vessels inside the tendon)
