# Supplementary material for: Subacromial bursa augmentation in arthroscopic rotator cuff repair: Clinical and doppler ultrasound assessment—A preliminary study
Source: J Exp Orthop. 2026 May 12;13(2):e70714. doi: 10.1002/jeo2.70714 (PMC13162131; doi:10.1002/jeo2.70714)
Supplement: Supplementary file 2 — AdditionalFile2. [file JEO2-13-e70714-s001.docx]

**ADDITIONAL FILE 2**

Ultrasonographic outcomes in the bursa augmentation group (Group B) and control group (Group C) at 3 weeks, 6 weeks, 3 months, and 6 months of follow-up.

|  | | | | | | | |
| --- | --- | --- | --- | --- | --- | --- | --- |
|  | **Group B** | | | **Group C** | | |  |
| Parameters | n | Mean (SD) | Median (range) | n | Mean (SD) | Median (range) | p-value |
| **Neovascularization Score (0-5) ROI: Bursa** | | | |  |  |  |  |
|  |  |  |  |  |  |  |  |
| 3 weeks | 17 | 1 (1) | 1 (0 to 3) | 10 | 1 (1) | 0 (0 to 4) | 0.245 |
| 6 weeks | 19 | 1 (1) | 0 (0 to 3) | 10 | 0 (1) | 0 (0 to 1) | 0.409 |
| 3 months | 19 | 1 (1) | 0 (0 to 3) | 10 | 0 (0) | 0 (0 to 1) | 0.304 |
| 6 months | 20 | 0 (0) | 0 (0 to 1) | 9 | 0 (0) | 0 (0 to 1) | 0.565 |
| **Neovascularization Score (0-5) ROI: Insertion** | | | |  |  |  |  |
|  |  |  |  |  |  |  |  |
| 3 weeks | 17 | 0 (0) | 0 (0 to 1) | 10 | 0 (0) | 0 (0 to 1) | 0.155 |
| 6 weeks | 19 | 1 (1) | 0 (0 to 4) | 10 | 0 (0) | 0 (0 to 0) | **0.032** |
| 3 months | 19 | 0 (1) | 0 (0 to 3) | 10 | 0 (0) | 0 (0 to 1) | 0.623 |
| 6 months | 20 | 0 (1) | 0 (0 to 2) | 9 | 0 (0) | 0 (0 to 1) | 1.00 |
| **Pain at rest (0=min, 10=max)** | | | | | | | |
| 3 weeks | 18 | 2 (2) | 2 (0 to 7) | 10 | 3 (2) | 4 (1 to 6) | 0.189 |
| 6 weeks | 18 | 1 (1) | 0 (0 to 4) | 10 | 3 (2) | 2 (0 to 6) | **0.013** |
| 3 months | 20 | 2 (2) | 1 (0 to 7) | 10 | 3 (2) | 2 (0 to 7) | 0.078 |
| 6 months | 20 | 1 (1) | 0 (0 to 4) | 10 | 2 (3) | 1 (0 to 8) | 0.137 |
| **Pain during activities (0=min, 10=max)** | | | | | | | |
| 6 weeks | 18 | 3 (2) | 1 (0 to 6) | 10 | 5 (2) | 6 (2 to 8) | 0.006 |
| 3 months | 20 | 3 (2) | 2 (0 to 9) | 10 | 5 (2) | 4 (3 to 8) | **0.009** |
| 6 months | 20 | 3 (2) | 2 (0 to 8) | 10 | 4 (3) | 3 (0 to 8) | 0.546 |
| **Supraspinatus tendon thickness (mm)** | | | | | | | |
| 3 weeks | 17 | 6 (1) | 6 (5 to 8) | 10 | 6 (1) | 6 (4 to 8) | 0.597 |
| 6 weeks | 19 | 6 (1) | 6 (5 to 8) | 10 | 5 (1) | 5 (4 to 7) | 0.103 |
| 3 months | 19 | 6 (1) | 6 (4 to 8) | 10 | 5 (1) | 5 (4 to 7) | **0.029** |
| 6 months | 19 | 6 (1) | 6 (4 to 8) | 9 | 5 (1) | 5 (3 to 5) | **0.007** |
| SD = standard deviation; p-value = Two-sample Wilcoxon rank-sum (Mann-Whitney) test for continuous variables | | | | | | | |
